# Structural and electronic properties of H_2_, CO, CH_4_, NO, and NH_3_ adsorbed onto Al_12_Si_12_ nanocages using density functional theory

**DOI:** 10.3389/fchem.2023.1143951

**Published:** 2023-02-16

**Authors:** Liu-Kun Li, Yan-Qiu Ma, Kang-Ning Li, Wen-Li Xie, Bin Huang

**Affiliations:** ^1^ Ningxia Key Laboratory of Intelligent Sensing for the Desert Information, School of Physics and Electronic-Electrical Engineering, Ningxia University, Yinchuan, China; ^2^ Basic Education Department, Guangdong Ocean University, Yangjiang, China; ^3^ Enviromental Monitoring Site of Ningxia Ningdong Energy and Chemical Industry Base, Yinchuan, China

**Keywords:** nanocage, Al_12_Si_12_, equilibrium geometries, stability, electronic properties

## Abstract

In this study, the adsorption of gases (CH_4_, CO, H_2_, NH_3_, and NO) onto Al_12_Si_12_ nanocages was theoretically investigated using density functional theory. For each type of gas molecule, two different adsorption sites above the Al and Si atoms on the cluster surface were explored. We performed geometry optimization on both the pure nanocage and nanocages after gas adsorption and calculated their adsorption energies and electronic properties. The geometric structure of the complexes changed slightly following gas adsorption. We show that these adsorption processes were physical ones and observed that NO adsorbed onto Al_12_Si_12_ had the strongest adsorption stability. The *E*_g_ (energy band gap) value of the Al_12_Si_12_ nanocage was 1.38 eV, indicating that it possesses semiconductor properties. The *E*_g_ values of the complexes formed after gas adsorption were all lower than that of the pure nanocage, with the NH_3_–Si complex showing the greatest decrease in *E*_g_. Additionally, the highest occupied molecular orbital and the lowest unoccupied molecular orbital were analyzed according to Mulliken charge transfer theory. Interaction with various gases was found to remarkably decrease the *E*_g_ of the pure nanocage. The electronic properties of the nanocage were strongly affected by interaction with various gases. The *E*_g_ value of the complexes decreased due to the electron transfer between the gas molecule and the nanocage. The density of states of the gas adsorption complexes were also analyzed, and the results showed that the *E*_g_ of the complexes decreased due to changes in the 3p orbital of the Si atom. This study theoretically devised novel multifunctional nanostructures through the adsorption of various gases onto pure nanocages, and the findings indicate the promise of these structures for use in electronic devices.

## 1 Introduction

Since Kroto et al. (1985) discovered (C_60_) fullerene, nanomaterials have gradually become a popular research topic due to their unique properties and wide range of applications (Goroff, 1996; Diederich and Gómez-López, 1999; Kataura et al., 2002; Martín, 2006). Instead of using only C to construct fullerenes, researchers have turned to fullerenes made from other elements, especially elements from group Ⅲ of the periodic table and the group V elements that are adjacent to C. These fullerenes include B_12_N_12_, Al_12_N_12_, B_12_P_12_, and Al_12_P_12_ (Beheshtian et al., 2012a; Beheshtian et al., 2012b; Beheshtian et al., 2012c; Rad and Ayub, 2016; Hussain et al., 2020a). In addition, researchers have used elements from groups Ⅱ and Ⅵ to construct similar fullerene structures, such as Be_12_O_12_, Mg_12_O_12_, Ca_12_O_12_, and all-boron fullerene (Kakemam and Peyghan, 2013; Zhai et al., 2014; Rezaei-Sameti and Abdoli, 2020; Ahsan et al., 2021). Other fullerenes are made from transition elements and oxygen element (de Oliveira et al., 2015). Nanocages with the general formula (XY)_12_, where *n* is the number of atoms, are more popular among researchers because they are more stable (Strout, 2000). Many types of nanostructures exist, among which inorganic nanostructures have attracted the attention of researchers due to their extremely high stability and asymmetric charge distribution (Dai, 2002; Tasis et al., 2006; Bindhu et al., 2016). The study of fullerene structures is a critical branch of nanotechnology because of their applications in electronic devices, special materials, and environmental processes. Shakerzdeh et al. (2015) studied the properties of alkali metals (Li, Na, and K) interacting with Be_12_O_12_ and Mg_12_O_12_ nanoclusters. Doping with alkali metals can significantly improve the non-linear optical response of nanocages. Beheshtian et al. (2012c) have studied the adsorption of NO and CO by Al_12_N_12_. Due to the different changes in the *E*_g_ (energy band gap) of NO and CO when adsorbed, resulting in different changes in electrical conductivity, Al_12_N_12_ clusters may selectively detect NO molecules when CO molecules are present. Vergara-Reyes et al. (2021) studied the adsorption of NO on the C_36_N_24_ fullerene, providing a possible carrier/protector of nitric oxide and thus fulfill its correct biological functions. Escobedo et al. (2019) conducted the effect of chemical order on the structural and physicochemical properties of B_12_N_12_ fullerene. Bautista Hernandez et al. (2022) found that the (TiO_2_)_19_ cluster is a good candidate for storing various gases, and can also be used as a hydrogen storage device.

For China in particular and the rest of the world in general, fossil fuel use must be scaled back, and hydrogen, which has the highest calorific value per unit mass of fuels and produces only water during combustion, is an attractive alternative (Yilanci et al., 2009; Züttel et al., 2010; Mazloomi and Gomes, 2012). Technologies for capturing pollutants and greenhouse gases from the atmosphere will also play a key role in our fight against climate change (Cazorla-Amorós et al., 1996; Zhang et al., 2008; Mastalerz et al., 2011).

Oku et al. (2004) synthesized the first inorganic nanocages in 2004 before the later emergence of B_12_N_12_, Al_12_N_12_, B_12_P_12_, and Al_12_P_12_ nanocages. Yarovsky and Goldberg, (2005) studied the adsorption of H_2_ by Al_13_ clusters using DFT (density functional theory). Thereafter, Felício-Sousa et al. (2021) examined the adsorption properties of H_2_, CO, CH_4_, and CH_3_OH on Fe_13_, Co_12_, Ni_13_, and Cu_13_ clusters using an *ab initio* investigation. Tan et al. (2022) studied clusters of different numbers of Al atoms. Yang et al. (2018) predicted the properties of electron redundant Si_n_N_n_ fullerenes. Metal oxide nanocages are also a popular area of research; Otaibi *et al.* conducted a theoretical study on the adsorption of glycoluril by Mg_12_O_12_ nanocages (Al-Otaibi et al., 2022). Some nanocages exhibit unique electronic properties after doping with alkali metals, and these have also been widely studied (Ahsin and Ayub, 2021). We replaced the N atoms in Al_12_N_12_ with Si atoms to investigate a more diverse range of materials than those in the literature. In this study, we adopted DFT to analyze the properties of Al_12_Si_12_, such as its stability, after the adsorption of CH_4_, CO, H_2_, NO, and NH_3_.

## 2 Computational methodology

An Al_12_Si_12_ nanocage was selected as the model adsorbent. Geometry optimization was performed using hybrid functional DFT (B3LYP) (Becke, 1993) implemented in Gaussian 09 (Frisch et al., 2004). B3LYP is a suitable and widely accepted functional for nanoclusters (Chen et al., 2009; Hussain et al., 2020b). The mixed basis set 6-31G (d, p) was used. Gas molecules CH_4_, CO, H_2_, NO, and NH_3_ were adsorbed onto Al_12_Si_12_ nanocages using the same method. Two adsorption sites on the Al_12_Si_12_ nanocage were considered. Vibration frequencies were also calculated at equivalent levels to verify that all stationary points corresponded to true minima on the potential energy surface. Geometry optimization was conducted, and *D*_ads_ (distance of adsorption), *E*_ads_ (adsorption energy), *E*_HOMO_ (energy of the highest occupied molecular orbital), *E*_LUMO_ (energy of lowest unoccupied molecular orbital), DOS (density of states), and *Q*_T_ (Mulliken charge transfer) were calculated to determine the adsorption mechanism.

The stability of the Al_12_Si_12_ nanocage was examined in terms of *E*_coh_ (cohesive energy), which can be determined by calculating the average energy difference of each atom before and after bonding using the following equation:

$$E_{coh}=\frac{E_{total}-12\left(E_{Al}+E_{Si}\right)}{24}$$

where *E*_Al_ and *E*_Si_ are the energies of non-interacting Al and Si atoms, respectively, and *E*_total_ is the energy of the Al_12_Si_12_ nanocage.

*E*_ads_ is defined as follows:

$$E_{ads}=E_{adsorbate@nanocage}-E_{adsorbate}-E_{nanocage}$$

Here, *E*_adsorbate@nanocage_, *E*_adsorbate_, and *E*_nanocage_ are the total energies of an adsorbate adsorbed onto the pure nanocage, of the adsorbate, and of the pure nanocage, respectively. A negative value of *E*_ads_ corresponds to exothermic adsorption. The more negative the adsorption value, the stronger the adsorption capacity. The DOS was generated in Multiwfn (O'Boyle et al., 2008).

## 3 Results and discussion

### 3.1 Geometrical characteristics

#### 3.1.1 Al_12_Si_12_ structure

**FIGURE 1:**
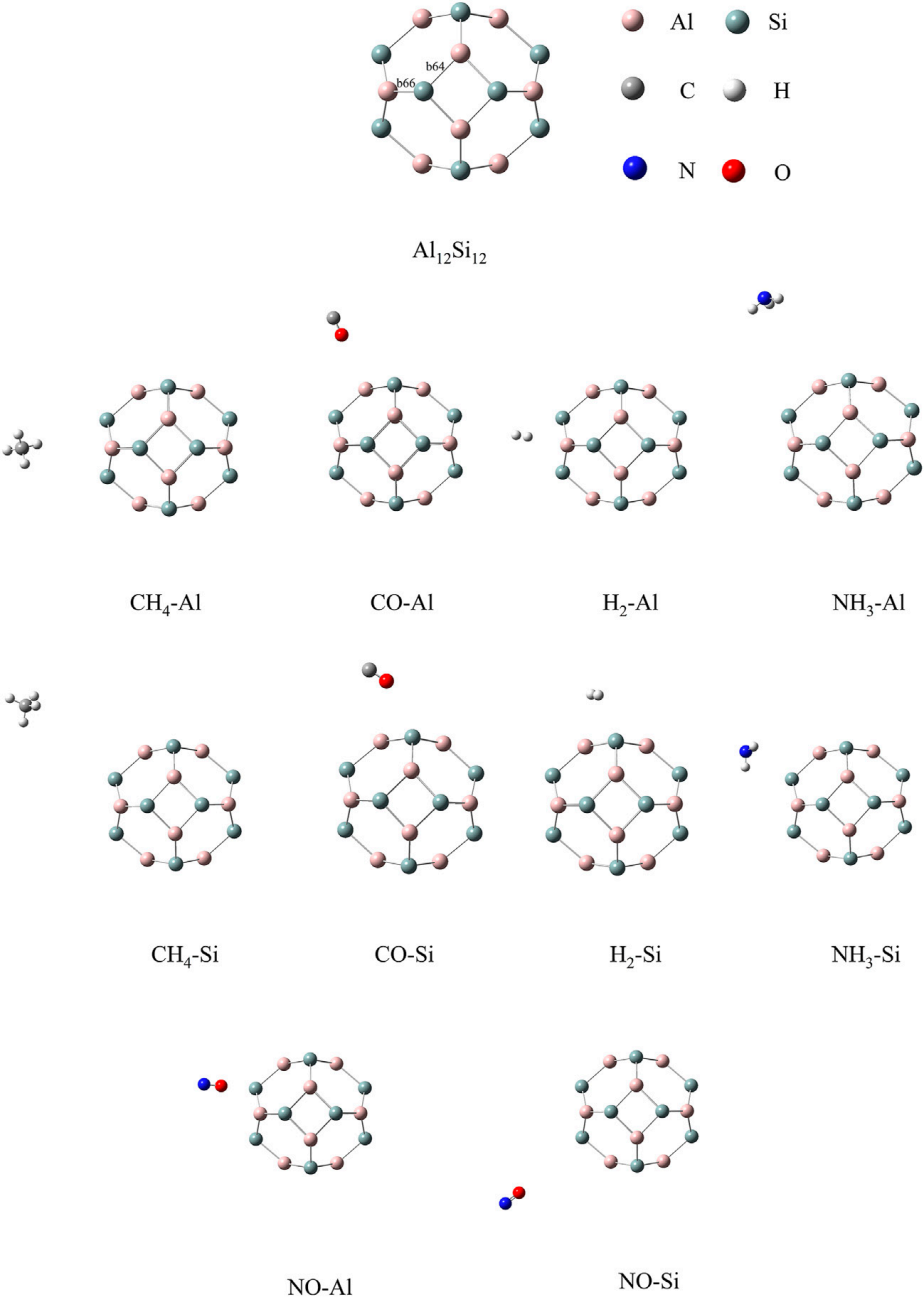
Stable configurations of nanocage complexes.

The optimized structures of the bare nanocage comprising eight hexagons and six tetragon rings are given in Figure 1. In this nanocage, two non-equivalent bonds exist: one between the tetragon and hexagon ring, and the other between the two hexagonal rings, represented as *b*_64_ and *b*_66_, respectively. The lengths of *b*_64_ and *b*_66_ are 2.47 and 2.43 Å, respectively. The calculated *E*_coh_ of Al_12_Si_12_ is −3.11 eV with zero point energy (ZPE) correction (Buelna-García et al., 2022). The calculated *E*_coh_ of each atom is closer to the reported *E*_coh_ of the most stable inorganic analogue, B_12_N_12_. The experimental and theoretical reported *E*_coh_ of B_12_N_12_ are −4.00 (Pokropivny et al., 2000) and −6.06 eV (Pokropivny and Ovsyannikova, 2007), respectively. As indicated by these results, the Al_12_Si_12_ nanocage is highly stable.

#### 3.1.2 Al_12_Si_12_–gas molecule structure

CH_4_, CO, H_2_, NO, and NH_3_ were selected as the target adsorbates. We performed sufficient structural optimization of the Al_12_Si_12_ nanocage containing CH_4_, CO, H_2_, NO, and NH_3_ molecules. For each gas molecule, two different adsorption sites above the Al and Si atoms on the nanocage surface were considered. Each gas is discussed with respect to adsorption at each of the two nanocage positions. The bond length (*b*_64_ and *b*_66_), *D*_ads_ value (defined as the center to center distance between the atoms of the nanocage and gas molecule that are closest to each other), and *E*_ads_ value are listed in Table 1. According to previous studies, the threshold *E*_ads_ at which physisorption becomes chemisorption is approximately 23.00 kcal/mol (1.00 eV) (Jouypazadeh and Farrokhpour, 2018; Nagarajan and Chandiramouli, 2019; Swetha et al., 2020). *D*_ads_ must be < 2.00 Å for chemisorption to occur (Contreras et al., 2014). As shown in Table 1, the adsorption processes were all physical, and NO adsorbed above the Al atom of the nanocage had the strongest adsorption stability.

**TABLE 1:** Bond length (*b*_66_ and *b*_64_), distance of adsorption (*D*_ads_) and energy of adsorption (*E*_ads_).

Complexes	*b*_66_ (Å)	*b*_64_ (Å)	*D*_ads_ (Å)	*E*_ads_ (kcal/mol)	Complexes	*b*_66_ (Å)	*b*_64_ (Å)	*D*_ads_ (Å)	*E*_ads_ (kcal/mol)
Al_12_Si_12_	2.43	2.47	—	—	Al_12_Si_12_	2.43	2.47	—	—
CH_4_-Al	2.47	2.43	5.76	−1.00	CH_4_-Si	2.47	2.43	7.36	−1.02
CO-Al	2.47	2.43	4.51	−1.05	CO-Si	2.47	2.43	4.39	−0.96
H_2_-Al	2.47	2.43	4.52	−12.37	H_2_-Si	2.47	2.43	3.92	−12.34
NO-Al	2.47	2.43	3.94	−15.35	NO-Si	2.47	2.43	5.58	−15.18
NH_3_-Al	2.47	2.43	6.11	−0.55	NH_3_-Si	2.47	2.42	3.48	−1.23

The calculated stable configurations (local minima) are summarized in Figure 1. When the gas was adsorbed onto the Al or Si atom of the Al_12_Si_12_ nanocage, slight local structural deformation of both the molecule and the nanocage occurred. The *b*_66_ and *b*_64_ bonds and angles remained almost unchanged. The adsorption that induced greatest change was that of NH_3_ onto the Si atom; it changed the lengths of *b*_66_ and *b*_64_ by 0.005 and 0.006 Å, respectively. The smallest change was caused by the adsorption of NO onto the Al atom; it changed the lengths of *b*_66_ and *b*_64_ by 0.0003 and 0.0204 Å, respectively.

In the Al atom adsorption group, the *E*_ads_ of CH_4_–Al, CO–Al, NH_3_–Al, NO–Al, and H_2_–Al were −1.00, −1.05, −0.55, −15.35, and −12.37 kcal/mol, respectively. The adsorption stability of the NO and H_2_ analytes was much better than that of the others. The *D*_ads_ values of NO–Al and H_2_–Al were 3.94 and 4.52 Å, respectively. For NO–Al, the N–O bond length increased from 1.06 Å in isolated NO to 1.16 Å in the adsorbed state. When Al_12_Si_12_ adsorbed H_2_, the H–H bond length increased from 0.6 Å in isolated H_2_ to 0.74 Å in the adsorbed state. The *D*_ads_ between the gas molecule and the Al atom of the nanocage were 5.76 and 4.51 Å for CH_4_–Al and CO–Al, respectively. The C–H bonds of CH_4_–Al and the C–O bond of CO–Al also became slightly longer. The lengths of the four C–H bonds of CH_4_–Al increased by 0.02269, 0.02286, 0.02290, and 0.02313 Å. The length of the C–O bond of CO–Al increased by 0.02 Å. The *D*_ads_ of NH_3_–Al was 6.11 Å. The length of the three N–H bonds in the NH_3_ molecule increased by 0.015636, 0.01599, and 0.01542 Å. Among these complexes, the three N–H bonds of NH_3_–Al had the smallest increase in length.

In the Si atom adsorption group, the *E*_ads_ of CH_4_–Si, CO–Si, NH_3_–Si, NO–Si, and H_2_–Si were −1.02, −0.96, −1.23, −15.18, and −12.34 kcal/mol, respectively. The *D*_ads_ values of CH_4_–Si, CO–Si, NH_3_–Si, NO–Si, and H_2_–Si were 7.36, 4.39, 3.48, 5.58, and 3.92 Å, respectively. In the Si atom adsorption group of complexes, both nanocages and gas molecules changed slightly. The length of the four C–H bonds increased by 0.02253, 0.02268, 0.02284, and 0.02315 Å. The length of the C–O bond increased by 0.02214 Å. The three N–H bonds increased in length by 0.01504, 0.01483, and 0.01485 Å. The lengths of the N–O bond and H–H bond increased by 0.09682, and 0.14372 Å, respectively. Among these complexes, the lengths of the three N–H bonds of NH_3_–Si increased by the least, but they increased by more than those of the three N–H bonds of NH_3_–Al in the Al group.

### 3.2 Electronic properties

#### 3.2.1 Mulliken charge and energy band gap analysis

Detailed information including the *E*_HOMO_ and *E*_LUMO_, *Q*_T_, and the △*E*_g_ (change in, *E*_g_ of nanocage upon adsorption) values is listed in Table 2.

**TABLE 2:** The Energy of HOMO (*E*_HOMO_), energy of LUMO (*E*_LUMO_), energy band gap (*E*_g_), change of *E*_g_ of nanocage upon the adsorption process (△*E*_g_), Mulliken charge transfer (Q_T_).

Complexes	*E*_LUMO_ (eV)	*E*_HOMO_ (eV)	*E*_*g*_ (eV)	△*E*_*g*_ (eV)	*Q*_T_ (|e|)	Complexes	*E*_LUMO_ (eV)	*E*_HOMO_ (eV)	*E*_*g*_ (eV)	△*E*_*g*_ (eV)	*Q*_T_ (|e|)
Al_12_Si_12_	−3.61	−4.99	1.38	—	—	Al_12_Si_12_	−3.61	−4.99	1.38	—	—
CH_4_-Al	−3.69	−5.00	1.31	−0.06	−0.0022	CH_4_-Al	−3.69	−5.00	1.31	−0.07	−0.0008
CO-Al	−3.67	−4.97	1.30	−0.08	0.0480	CO-Al	−3.69	−5.00	1.31	−0.07	0.0001
H_2_-Al	−3.69	−4.99	1.30	−0.08	0.0197	H_2_-Al	−3.69	−5.00	1.31	−0.07	0.0224
NO-Al	−3.70	−4.99	1.29	−0.09	0.0190	NO-Al	−3.68	−5.00	1.32	−0.06	0.0021
NH_3_-Al	−3.74	−5.05	1.31	−0.07	−0.0021	NH_3_-Al	−3.76	−4.99	1.23	−0.15	0.1177

In the Al atom adsorption group, the Mulliken charge analysis shows that the number of transferred electrons in CH_4_–Al, CO–Al, NH_3_–Al, NO–Al, and H_2_–Al were −0.0020, −0.0480, −0.0021, 0.0190, and 0.0197 |e|, respectively. The *E*_g_ of CH_4_–Al, CO–Al, NH_3_–Al, NO–Al, and H_2_–Al are 1.31, 1.30, 1.31, 1.29, and 1.30 eV, respectively. The *E*_g_ of these complexes was lower than that of the pure nanocage, and this decrease was largest in the NO complex, at 0.09 eV, and smallest in the CH_4_ complex, at 0.06 eV.

In the Si atom adsorption group, the Mulliken charge analysis demonstrated that the number of transferred electrons in CH_4_–Si, CO–Si, NH_3_–Si, NO–Si, and H_2_–Si were −0.0008, 0.0001, 0.1177, 0.0021, and 0.0224 |e|, respectively. The *E*_g_ of CH_4_–Si, CO–Si, NH_3_–Si, NO–Si, and H_2_–Si was 1.31, 1.31, 1.23, 1.31, and 1.31 eV, respectively. The *E*_g_ values of these complexes were also lower than that of the pure nanocage, with the NH_3_ complex exhibiting the greatest decrease, at 0.15 eV, and the NO complex exhibiting is the smallest decrease at 0.06 eV.

As demonstrated in Table 2, the *E*_LUMO_ values of these complexes were slightly lower than those of the pure nanocage, but their *E*_HOMO_ values did not vary much from those of the pure nanocage. The adsorption stability of the Si adsorption group of complexes is similar to that of the Al adsorption group. However, the adsorbates in the Si adsorption group transferred fewer electrons than the corresponding adsorbates in the Al adsorption group, except for the H_2_ and NH_3_ analytes. The N atom gained electrons, and the three H atoms lost electrons in the NH_3_–Al adsorption. However, the N atom and the three H atoms all lost electrons in the NH_3_–Si adsorption due to the difference in adsorption position. The adsorption stability of NH_3_–Si was higher than that of NH_3_–Al due to the short adsorption distance and the large number of transferred electrons in the NH_3_–Si adsorption.

#### 3.2.2 Frontier molecular orbitals and DOS

**FIGURE 2:**
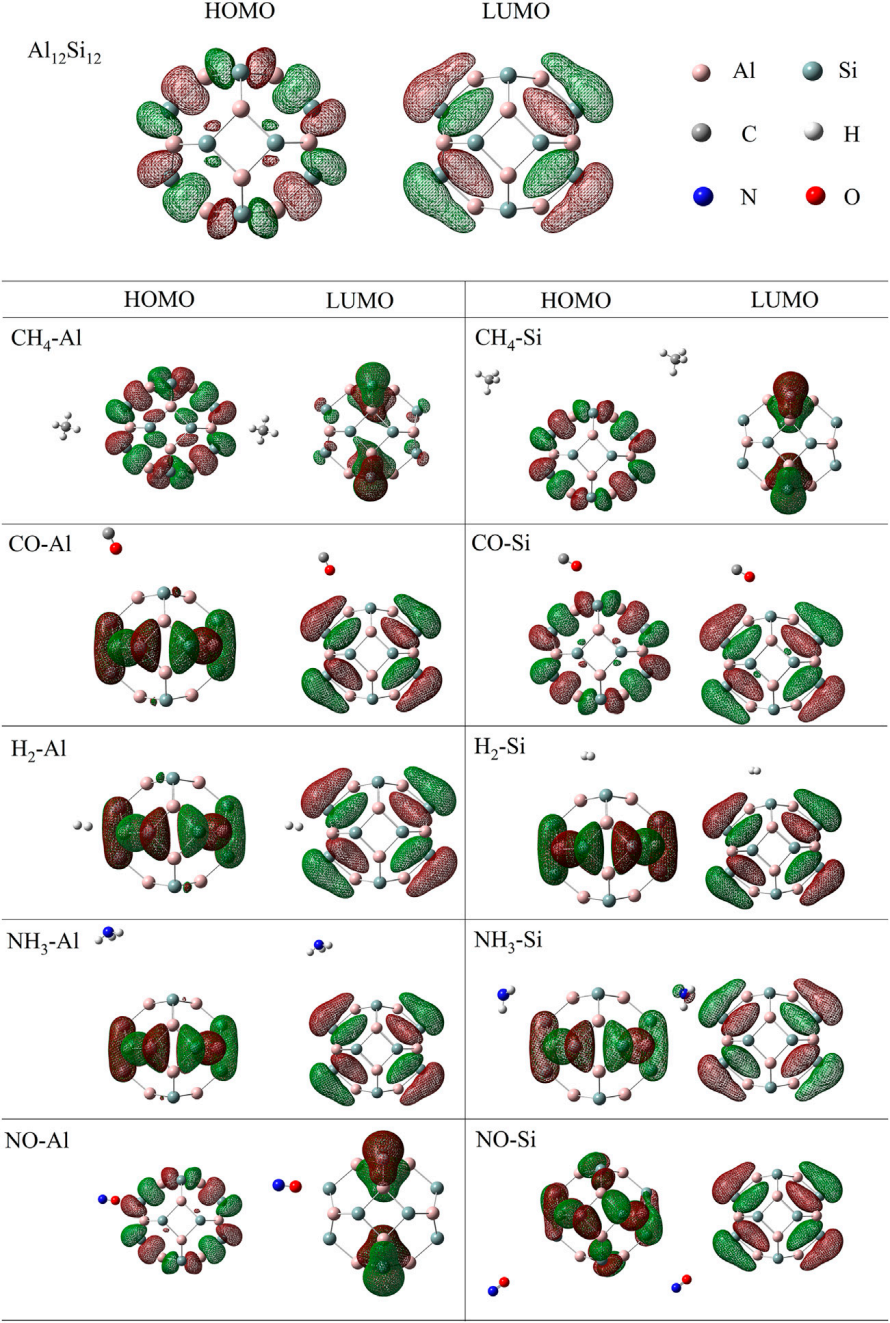
HOMO and LUMO distributions of nanocage complexes.

To further analyze the effect of gas molecules on the electronic properties of nanocages, the HOMO (highest occupied molecular orbital), LUMO (lowest unoccupied molecular orbital) and DOS of isolated and complexed nanocages were analyzed. The HOMO and LUMO distributions of Al_12_Si_12_ from a vertical perspective are shown in Figure 2. The HOMO primarily serves as an electron donor, and the LUMO primarily serves as an electron acceptor (Dai, 2002). For the HOMO of the pure Al_12_Si_12_ nanocage, the positive and negative areas alternate between Al and Si atoms (in Figure 2 the red area is negative, the green area is positive). However, for the LUMO of the pure Al_12_Si_12_ nanocage, the positive and negative areas alternated between the inside and outside of the nanocage and the HOMO and LUMO were distributed on the same plane. The obtained frontier molecular orbital energies (*E*_HOMO_ and *E*_LUMO_) and the calculated, *E*_g_ value of the nanocages are shown in Table 2. The *E*_g_ of Al_12_Si_12_ was approximately 1.38 eV, demonstrating its semiconductor properties.

The HOMO and LUMO plots of the complexes are displayed in Figure 2. The lower the *E*_g_ value, the more easily a molecule was excited. In the Al adsorption group, the HOMO and LUMO of complexes were almost unchanged. After gas adsorption, the distribution positions of the HOMO and LUMO changed from being on the same plane to being perpendicular to each other. In the Si adsorption group, the HOMO and LUMO of CO–Si remained on the same plane after adsorption. This may be due to the low number of electron transfers. In NH_3_–Si, the LUMO distribution was in the NH_3_ molecule. This may be because NH_3_–Si has the shortest adsorption distance and the greatest charge transfer. In NO–Si, part of the HOMO is transferred to the quadrilateral ring opposite the top and bottom. This is most likely because the HOMO energy level of NO–Si is lower than that of NO–Al.

**FIGURE 3:**
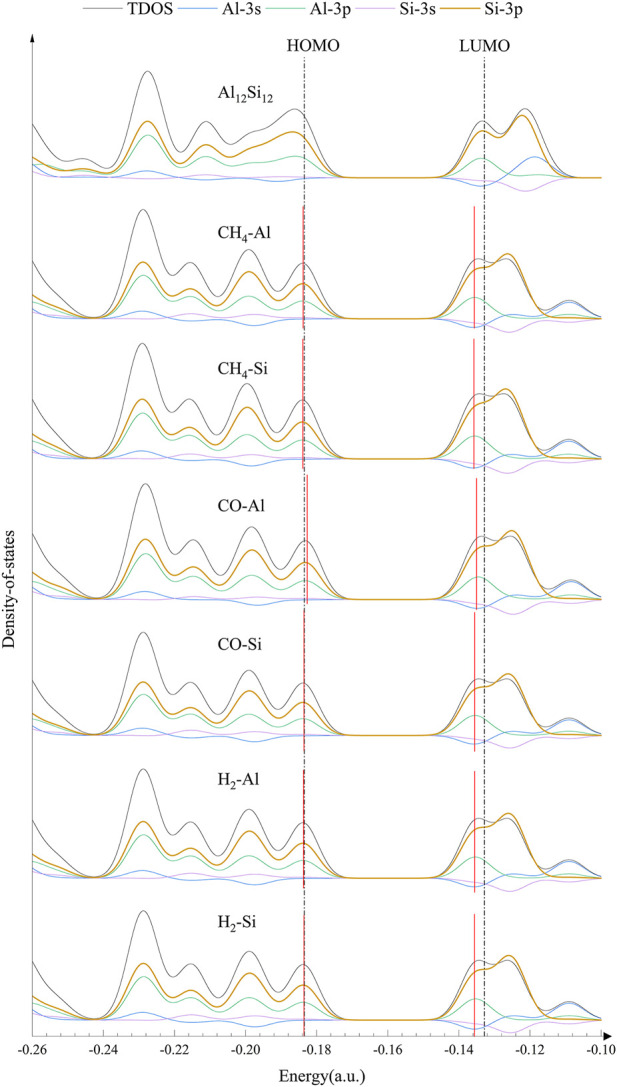
DOS (density of states) of nanocage complexes.

**FIGURE 4:**
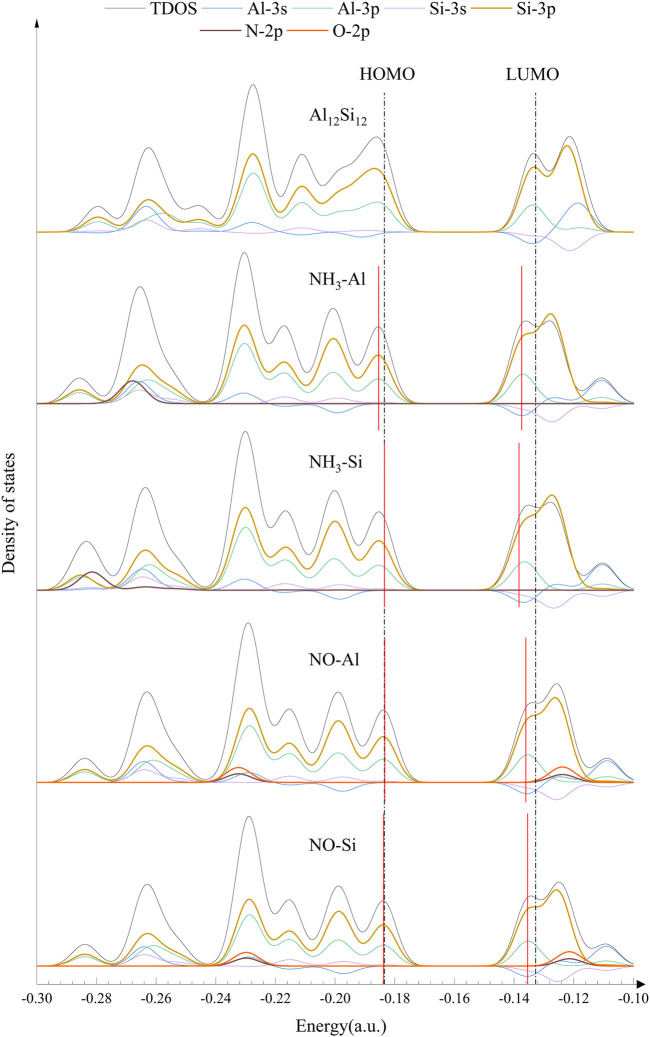
DOS (density of states) of nanocage complexes.

For further confirmation of the electronic behavior of these complexes, DOS analyses were performed (shown in Figures 3, 4). The orbitals in the lower energy region of Al_12_Si_12_ were mainly the 3p orbitals of the Si and Al atoms. At approximately −0.26 a.u., 3s orbitals of both the Al atoms and the Si atoms were maily present. The HOMO–LUMO energy level was composed mainly of the 3p orbitals of the Si and Al atoms, whereas the 3s orbitals of Al atoms were also present near the LUMO energy level.

The effect of the gas molecular orbital on the complex orbital when the nanocage adsorbed some gases was not significant (atomic orbitals that do not contribute to the total orbitals are not depicted in Figures 3, 4). We divided the complexes into two groups: The first including gas molecular orbitals that did not contribute to the total orbitals of the complexes, and the second including the gas molecular orbitals that contributed to the total orbitals of the complexes. In the first group, after gas adsorption, a new peak at 0.2 a.u. appeared, resulting in a slight decrease in the HOMO of the complexes (except for CO–Al). In addition, due to the adsorption of the gas molecule, the 3s orbital of the Al atom produced a small peak at −0.12 a.u. (shown by the blue curve), causing the peak at −0.11 a.u. to shift to the right slightly. This may be the reason for the decrease in LUMO energy level. In the second group, after gas adsorption, a new peak also appeared at 0.2 a.u.. The 3p orbital of the N atom in NH_3_–Al peaked at −0.27 a.u., whereas it peaked at −0.28 a.u. in NH_3_–Si (depicted by the brown curve). The 3p orbitals of the N and O atoms in NO–Al and NO–Si had peaks at −0.23 and −0.12 a.u. (the brown curve and the orange curve), respectively. Moreover, the peak value of the 3p orbital of the O atom was greater than that of the N atom. However, compared with the first group, the new peaks in the second group caused almost no decrease in HOMO and LUMO energy levels. The peak generated at 0.2 a.u. of the 3p orbital of the Si and Al atoms was the main reason for the reduction in HOMO energy level.

## 4 Conclusion

DFT calculations were performed to investigate the equilibrium geometries, stabilities, and electronic properties of gases adsorbed onto Al_12_Si_12_ nanocages. The *E*_g_ value of Al_12_Si_12_ was 1.38 eV, demonstrating the semiconductor properties of the nanocage. We performed structural optimization of Al_12_Si_12_ nanocages with adsorbed CH_4_, CO, H_2_, NO, and NH_3_ molecules to study their adsorption energies, equilibrium geometry, and electronic properties. Two adsorption positions on the nanocage were studied. The adsorption energy of these complexes demonstrated that the adsorption of these gases by Al_12_Si_12_ nanocages is physical. Our findings reveal that the stabilities of the complexes are as follows: Al_12_Si_12_–NO > Al_12_Si_12_–H_2_ > Al_12_Si_12_–NH_3_ >Al_12_Si_12_–CH_4_ >Al_12_Si_12_–CO. The geometric structure of the nanocage changed slightly following adsorption of molecules. The *E*_g_ of the complexes was lower than that of the pure nanocage due to the electron transfer between the gas molecules and the nanocage. The more electrons were transferred, the greater the decrease in *E*_g_. Except for the 3p orbitals of the N and O atoms, the orbitals of the gas molecules did not contribute to the total orbitals of the complexes. The 3p orbitals of the Si atoms in the Al_12_Si_12_ nanocage are the main reason for the change in HOMO–LUMO energy levels detected.

## Data Availability

The original contributions presented in the study are included in the article/supplementary material, further inquiries can be directed to the corresponding author.
